# Efficacy of telemedicine for persons with type 1 diabetes during Covid19 lockdown

**DOI:** 10.1038/s41387-020-00147-8

**Published:** 2021-01-05

**Authors:** Federico Boscari, Sara Ferretto, Ambra Uliana, Angelo Avogaro, Daniela Bruttomesso

**Affiliations:** Department of Medicine, Unit of Metabolic Disease, University of Padova, 35128 Padova, Italy

**Keywords:** Type 1 diabetes, Patient education

## Abstract

**Background:**

Starting March 2020 the Italian Government imposed a lockdown to limit the spread of SARS-CoV-2. During lockdown outpatient visits were limited and telemedicine (TM) was encouraged.

**Methods:**

We retrospectively analyzed data from continuous or flash glucose monitoring systems shared through different cloud systems during the lockdown by subjects with type 1 diabetes and compared data obtained 4 weeks before and 4 weeks after structured telephonic visit. Variables considered were mean glucose, time spent in target (70–180 mg/dl), hypoglycemia (<70 mg/dl) and hyperglycemia (>180 mg/dl), coefficient of variation, and length of sensor use.

**Results:**

During the 4 weeks following the telephonic visit there was an improvement of glycemic control, with a significant reduction of mean glucose values (161.1 before vs 156.3 mg/dl after, *p* = 0.001), an increase of the time spent in target (63.6 vs 66.3, *p* = 0.0009) and a reduction of time spent in hyperglycemia (33.4 vs 30.5, *p* = 0.002). No changes were observed regarding glucose variability, time spent in hypoglycemia, and length of sensor use. Similar results were observed in subjects treated with multiple daily injections or continuous subcutaneous insulin infusion.

**Conclusions:**

A structured telephonic visit appears to be an effective way to replace or integrate routine visits in particular conditions.

## Introduction

In December 2019, an epidemic of pneumonia related to a new strain of Coronavirus developed in the Chinese province of Wuhan. The infection is caused by the severe acute respiratory syndrome coronavirus 2 (SARS-CoV-2), caries a high mortality risk and is now known as coronavirus disease 2019 (COVID- 19)^1,2^.

Starting February 2020, COVID19 hit abruptly North Italy that quickly became the area in the world most severely affected by the infection. To limit the spread of the epidemic at the end of February, the Italian government imposed a series of restrictions which led to the complete closure of any non-urgent work and commercial activity^3^ arriving, during March and April 2020 to a complete lockdown. During the lockdown all citizens were invited to stay home and hospitals canceled all non-urgent activities, including visits of people with diabetes^4,5^.

To control glucose levels, persons with type 1 diabetes (T1D) use glucometers or devices for continuous glucose monitoring, that both allow the sharing of data with their clinicians through dedicated cloud platforms, indeed clinicians have taken advantage of this opportunity to replace or supplement regular visits particularly in subjects using continuous glucose monitoring (CGM) or Flash glucose Monitoring (FGM) systems^6–9^.

CGM and FGM data can be visualized through receivers or Smartphone apps and these data can be uploaded into a cloud-system if the receiver is connected to a website or can be transmitted to a smartphone app in real time. Different CGM and FGM systems have developed specific web platforms that allow clinicians and patients to interact both during visits and by remote (telemedicine).

It has been demonstrated that telemedicine improves some psychosocial outcomes in young adults with diabetes^10^, but there are few data regarding its efficacy in glycemic control. Very recently some evidence has been published about the role of telemedicine in T1D in particular populations or in special situations, for example in new diagnosed T1D^11–13^.

There are few data regarding the efficacy of telemedicine in persons with T1D. Aim of the study was to evaluate the role of telemedicine in patients with TID using CGM or FGM under the extreme circumstances brought about by COVID-19.

## Methods

This is a monocentric observational retrospective study conducted at the Unit of Metabolic Disease of the University of Padova, between 9 March and 11 May 2020, when regular visits were replaced by a structured phone colloquium (Telemedicine Visit, TM).

We enrolled all consecutive patients with T1D, using a CGM or FGM and who performed a TM. Enrolled patients have been using FGM or CGM for more than 6 months and actively shared active data with our clinic through dedicated platforms. The platforms used were Libreview (Abbott Diabetes Care) for FGM data (FreeStyle Libre, Abbott Diabetes Care) www.libreview.com, Dexcom Clarity^14^ (Dexcom, San Diego, CA) for Dexcom sensors (DexcomG5 and Dexcom G6), and Eversense DMS (Eversense, Senseonics) for implantable sensor. Subjects treated with Multiple daily injection (MDI) and continuous subcutaneous insulin infusion (CSII) were enrolled, with no restriction about metabolic control. Pregnant subjects were excluded because glycemic control could be influenced by different glycemic target required during pregnancy. Similarly, subjects switched from MDI to CSII in the previous 6 months were excluded to avoid changes in glucose control related to new therapy.

During TM the clinician evaluated glucose data accessible through platforms, and registered all blood and instrumental exams sent by email. Data were discussed with the subjects during TM and, at the end of the connection, a report was sent by email to the patient with changes in insulin dosage on the basis of the glucose trend.

We evaluated glucose control according to existing guidelines^15^. In particular, we evaluated mean glucose values, glucose management indicator (GMI, a new formula to estimate glycosylated Hemoglobin from mean glucose values)^16^, time spent in target range (% of time with glucose values between 70 and 180 mg/dl), in hypoglycemia (% of time with values below 70 mg/dl) or in hyperglycemia (% of time with glucose values above 180 mg/dl), and the coefficient of variation (CV) that is now considered the best indicator of glucose variability. Furthermore, we evaluated information on sensor use, defined as % of sensor use during the period. Regarding FGM use we collected data about number of scans performed during each period. All these data were automatically generated by the platform software of each device. Baseline metabolic control, expressed as mean of HbA1c values in the previous year, and anthropometric parameters were obtained from electronic records.

To evaluate TM efficacy, we considered glucose control during the 4 weeks before and the 4 weeks after TM. A separated analysis regarding differences between subjects treated with MDI and CSII was performed to investigate if glycemic control changes could be related with therapy.

The study was performed in accordance with the Helsinki Declaration of 1964, and its later amendments, and was in agreement with national regulations. The study was conceived as a retrospective data collection and notification of the study has been done to the local Ethical Committee.

### Statistical analysis

Continuous variables are presented as the mean ± standard deviation (SD) if normally distributed or as the median and interquartile range (IQR) if non-normally distributed. Categorical variables were presented as percentages. Comparison of variables recorded before and after TM visit was performed using the two-tail paired Student’s *t* test. Statistical significance was accepted at *p* < 0.05.

## Results

We enrolled 71 T1D (Table 1), mean age 41.9 years, with an acceptable metabolic control during the previous year (mean HbA1c 7.5%). Most of the subjects were in multi daily injections (MDI) therapy (54.9%). Patients on MDI were younger and with shorter diabetes duration than MDI subjects.

**Table 1:** Baseline characteristics of subjects.

Parameters	All	MDI	CSII
Patients, *n* (%)	71 (100)	39 (54.9)	32 (45.1)
Males, *n* (%)	32 (45.1)	21 (53.8)	11 (34.4)
Age (years), mean (SD)	41.9 (14.3)	36.6 (13.9)	48.2 (12.3)
Diabetes duration (years), mean (SD)	25.9 (13.8)	20.7 (13.8)	31.9 (11.3)
BMI (Kg/m^2^), mean (SD)	23.7 (3.7)	22.9 (3.5)	24.7 (3.6)
HbA1c (%), over the last year, mean (SD)	7.5 (0.9)	7.5 (1.0)	7.4 (0.6)
HbA1c (mmol/mol), over the last year, mean (SD)	58.3 (9.3)	59.1 (11.1)	57.7 (6.7)
FGM, *n* (%)	52 (73.2)	35 (89.7)	17 (53.1)
CGM, *n* (%)	19 (26.8)	4 (10.3)	15 (46.9)

*BMI* body mass index, *MDI* multiple daily injections, *CSII* continuous subcutaneous insulin infusion, *FGM* flash glucose monitoring, *CGM* continuous glucose monitoring.

Fifty two subjects used FGM and 19 used CGM, the majority of devices being from Dexcom (84.2% of CGM users).

Glucose control improved after TM. In fact during the 4 weeks after TM (Table 2) there was a significant reduction of mean glucose values (*p* = 0.001), an increase of the time spent in target (from 63.6% to 66.4%, *p* < 0.001) and a reduction of the time spent in hyperglycemia (from 33.4% to 30.5%, *p* = 0.001). No changes were observed in time spent in hypoglycemia and in CV. The improvement in glycemic control does not appear to be due to differences in the time of sensor use (Table 2), nor to the number of scans for subjects using FGM (11.4 vs 10.3 scan/day, *p* = 0.3).

**Table 2:** Glycemic control before and after telemedicine visit.

Parameter	4 weeks before TM	4 weeks after TM	*p*
Mean glucose (mg/dl), mean (SD)	161.1 (23.1)	156.3 (21.5)	0.001
GMI (%), mean (SD)	7.16 (0.56)	7.05 (0.53)	0.002
CV (%), mean (SD)	33.9 (4.8)	33.9 (5.5)	0.9
Time in target (%), mean (SD)	63.6 (15.3)	66.3 (15.1)	0.0009
Time in hypoglycemia (%), mean (SD)	3.0 (2.7)	3.2 (3.4)	0.6
Time in hyperglycemia (%), mean (SD)	33.4 (15.7)	30.5 (15.3)	0.002
Sensor use (% per period) mean (SD)	92.6 (14.3)	92.3 (15.1)	0.9

*GMI* glucose management indicator, *CV* coefficient of variation.

Considering MDI and CSII subjects separately, as shown in Table 3, we found similar effects of TM on glycemic control in both groups.

**Table 3:** Glycemic control before and after TM in MDI and CSII subjects.

Parameter	4 weeks before TM	4 weeks after TM	*P*
**Mean glucose (SD), mg/dl**			
MDI	163.7 (28.4)	159.3 (25.7)	0.05
CSII	157.9 (14.0)	152.6 (14.4)	0.003
**Coefficient of variation (%), mean (SD)**			
MDI	33.2 (5.5)	32.9 (5.0)	0.6
CSII	35.0 (4.1)	35.2 (6.1)	0.7
**Time in target (%), mean (SD)**			
MDI	61.5 (18.1)	64.7 (18.2)	0.008
CSII	66.1 (10.7)	68.4 (10.3)	0.05
**Time in hypoglycemia (%), mean (SD)**			
MDI	3.0 (2.6)	2.8 (2.6)	0.6
CSII	3.0 (2.9)	3.7 (4.2)	0.1
**Time in hyperglycemia (%), mean (SD)**			
MDI	35.5 (18.9)	32.6 (18.6)	0.03
CSII	30.8 (10.2)	27.9 (9.8)	0.01
**Sensor use (%), mean (SD)**			
MDI	90.4 (16.7)	90.4 (18.3)	0.9
CSII	95.2 (10.3)	94.6 (9.7)	0.8

Finally, we divided subjects in 2 groups, on the basis of TM visit date, to exclude that the impact on glycemic control was linked to the lockdown itself, as recently demonstrated^17^. We evaluated separately subjects that lived during lockdown both periods, before and after TM. This subgroup, composed by 30 subjects, had a similar improvement of glycemic control with a reduction of mean glucose from 160.2 to 154.4 (*p* = 0.01), an increase of time spent in target from 64.1 to 67.1 (*p* = 0.009), and a reduction of time spent in hyperglycemia (32.6 vs 29.6, *p* = 0.01).

## Discussion

This study analyzed feasibility and efficacy of remote glycemic control in T1D subjects, through a telephone visit. The Covid 19 emergency made this type of remote control necessary but once the emergency was over, this type of approach could be offered as an alternative or integration to the usual visit.

Obviously, the remote control is not intended to replace the visit, especially as regards education in the management of diabetes and use of technology.

We found that a structured telemedicine visit, which includes the discussion of glycemic data and the provision of written suggestions, had a positive impact in the glycemic control of T1D. We recently demonstrated that during lockdown glycemic control improved in patients who discontinued work^17^, probably because they were able to pay more attention to diabetes control. However, the improvement in glycemic control that we found after the telemedicine visit does not appear to be attributable to the lockdown since the telemedicine visit improved glucose control also in the subgroup that entirely lived in lockdown during both periods, before and after TM. Efficacy of TM is not related to type of insulin therapy since the improvement was similar in MDI and CSII subjects.

We have no data regarding T1D satisfaction, but it is reasonable to consider that this solution can be appreciated by patients. In fact, TM leads to a journey reduction for patients, with a reduction in the loss of working days.

This study has limitations. First, just patients using continuous blood glucose monitoring devices were evaluated.

Furthermore, we excluded people who did not use the data sharing platforms with the possibility of selecting persons most motivated and more bent to use new technologies.

Finally, the present conclusions about TM efficacy were reached through a short study regarding a small number of subjects. A short observation obviously leads to a lack of information about long-term metabolic control, like HbA1c.

This study shows that telemedicine can be a useful and effective support in the management of subjects with T1D. The strategy changes imposed by the COVID 19 emergency could open new avenues in the treatment of sick patients^18^.
